# The complete chloroplast genome sequence of *Bupleurum chinense* DC. (Apiaceae)

**DOI:** 10.1080/23802359.2019.1678427

**Published:** 2019-10-18

**Authors:** Fei Zhang, Zhenyu Zhao, Qingjun Yuan, Suiqing Chen, Luqi Huang

**Affiliations:** a Pharmacy College of Henan University of Chinese Medicine, Zhengzhou, PR China;; b National Resource Center for Chinese Meteria Medica, China Academy of Chinese Medical Sciences, Beijing, PR China

**Keywords:** *Bupleurum chinense*, chloroplast genome, phylogeny

## Abstract

*Bupleurum chinense* DC. is a traditional medicinal herb species widely distributed in most provinces of China. In this study, we constructed and annotated a complete circular chloroplast (cp) genome of *B. chinense*. The cp genome of *B. chinense* is 155,545 bp in length, including two inverted repeat (IR) regions of 26,305 bp, separated by a large single-copy (LSC) region of 85,430 bp and a small single-copy (SSC) region of 17,505 bp. The GC content of whole cp genome is 37.68%. The genome contains 113 different genes, including 79 protein-coding genes, 30 tRNA genes, and 4 rRNA genes. A maximum-likelihood phylogenomic analysis showed that *Bupleurum* formed a monophyletic group, and was sister to other groups of Apiaceae.

*Bupleurum chinense* DC. is a perennial plant in the Apiaceae family (Lin et al. 2008). The genus *Bupleurum* includes approximately 200 species, many of which have been pharmaceutically used for thousands of years, mainly in Asia and Europe (Pan 2006). In addition to the authentic species of *B. chinense*, there are more than 20 other species in *Bupleurum* also habitually utilized as Bupleuri Radix in some local areas. Knowing the high demand for Bupleuri Radix and the diversity of species that can be – both rightly and wrongly – used for this herb, the resources of *B. chinense* are very scarce (Liu et al. 2011). Here, we assembled the complete chloroplast (cp) genome sequence of *B. chinense* based on the next-generation sequencing method. The primary purpose of this study was to analyze the structure of the complete cp genome of *B. chinense* and to resolve the phylogenetic relationships of *B. chinense* and other species in Apiaceae.

Fresh samples of *B. chinense* was collected from Zhashui county (Shaanxi province, China; 109°20′27. 24″E, 33°48′13. 68″N). Voucher specimen was stored in the herbarium of Institute of Chinese Materia Medica (CMMI), China Academy of Chinese Medical Sciences. The specimen voucher number is 611026LY0126. Total genomic DNA was extracted from the dry materials using the mCTAB approach (Li et al. 2013).

Using the Illumina HiSeq Xten platform, a 400 bp (insertion size) double-ended library was constructed by splicing DNA. The readings from the paired-end were qualitatively assessed and assembled with SPAdes version 3.13.1 (Bankevich et al. 2012). Plann is used for initial annotation, Sequin for corrections (Huang and Cronk 2015). The complete cp genomic sequence annotated had been submitted to GenBank under the accession number of MN337347 for *B. chinense*.

The structure of cp genome of *B. chinense* was circular, and the size was 155,545 bp. It included two inverted repeat (IR) regions of 26,305 bp, separated by a large single-copy (LSC) region of 85,430 bp and a small single-copy (SSC) region of 17,505 bp. The whole cp genome GC content is 37.68%. There are 113 different genes in the cp genome of *B. chinense*, including 79 protein-coding genes, 30 tRNA genes, and 4 rRNA genes.

We chose 21 species within the family Apiaceae and *Hydrocotyle verticillata* (Araliaceae) as an outgroup to establish a phylogenetic tree by the maximum likelihood (ML) analysis using RAxML with 1000 bootstrap replicates (Stamatakis 2014). The phylogenetic tree showed that *B. chinense* and other species of *Bupleurum* formed a monophyletic group, and was sister to other groups of Apiaceae (Figure 1). The complete cp genome of *B. chinense* provided a lot of genetic information for species conservation and identification as well as the phylogenetic studies of Apiaceae.

**Figure 1.**
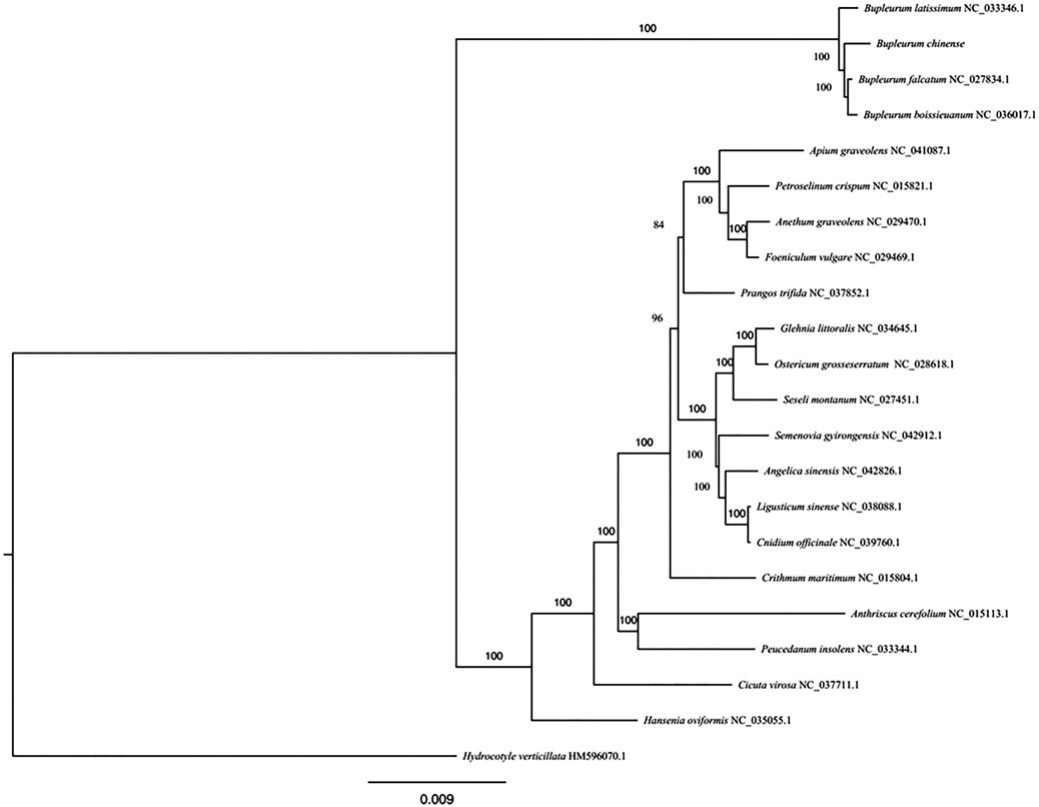
The best ML phylogeny recovered from 22 complete chloroplast sequences by RAxML. Bootstrap support values are given at each node.
